# The Employment of Dentists in the Army and Navy

**Published:** 1870-08

**Authors:** F. Y. Clark

**Affiliations:** Savannah, Ga.


### THE EMPLOYMENT OF DENTISTS IN THE ARMY AND NAVY.

BY F. Y. CLARK, M. D., D. D. S., SAVANNAH, GA.

"*Resolved:*—That this society instruct their delegates to the American and Southern Dental Association to request said Association to appoint Dentists in the army and navy of the United States."

This is a resolution passed by the Georgia State Dental Society at its annual meeting in Savannah, December 29th, 1869, and as a delegate from that society I find it my duty to bring the subject before this body for further consideration.

Perhaps no circumstance can more clearly testify to the increasing importance of the subject before us than a few extracts from some of the English journals. It is a well known fact that, as a nation, the teeth of the English are much better than ours; yet the far-seeing Medical Director General of the British Army saw the benefit that might be derived from the employment of a few Dentists in the army and hospitals; and therefore made the following recommendation, which is copied from his circular of 1857, in the May number of the *British Journal of Dental Science:*

"Among the improvements in conservative surgery, not the least remarkable and important is the successful application of mechanical ingenuity and skill, whereby the necessity formerly considered almost imperative for the extracting of teeth affected with caries has been in a great measure averted. Thus improved dental surgery has rendered the measure of extracting (formerly the rule) now the rarely occurring exception, and this better practice must be regarded as likely to exercise an influence by no means slight towards the improvement of the health of that portion of the community where means of recourse is being had to the aid which skillful dentistry supplies, for the preservation of the teeth. * * * I am, therefore, most anxious that all military medical officers should give the subject their best attention, as I am of opinion that a considerable gain to the service, besides comfort to individuals, would occur from the practice of dental surgery in the army, to a greater extent than that which has hitherto been obtained in military life.

"Dental surgery in the army will, I feel convinced, if carefully practiced, exercise not an unimportant influence on the efficiency of regiments; will obviate the necessity of having recourse to the forceps in numerous cases, and will add to the comfort and health of the soldiers."

Perhaps no better comment on the result of the above recommendation can be made than that which we find in the

British and foreign *Medico Chirurgical Review*, of January, 1860, three years after the above named circular was issued:

"Five years ago there were not more than two of the hospitals in London at which the necessity of a Dentist was recognized; at the present day so fully is this want admitted that there is no hospital, and scarcely a dispensary without such an officer."

In 1859 this subject was brought before the American Dental Convention and a committee appointed to memorialize Congress. The following is the resolution, as introduced by Dr. McKellops, of St. Louis:

"WHEREAS: Owing to the great inconvenience of the officers and soldiers in procuring competent Dentists, when necessary, and knowing the difficulty in which they are placed, being stationed at distant posts and places where it is impossible for a Dentist to visit, therefore

"*Resolved*, That this society appoint a committee of five for the purpose of memorializing Congress on the necessity of appointing Dentists to be attached to the regular army; and that we recommend the matter to the consideration of the general Government."

The resolution was adopted, and the committee appointed, but we have not been able to learn whether this committee took any step in the matter, or not. It may have been in connection with this that Jefferson Davis, when Secretary of War to the United States, made an effort to employ Dentists in the army and navy, and no doubt would have succeeded had he remained in office. From this time, until after the commencement of the war, we know of no farther effort being made. Shut in as we were from the North, and the rest of the world, we knew little of what was going on without. When the steamer "Water Witch" was captured, and the prisoners brought to Savannah, we learned there was a Dentist among the number; and that he had quite a supply of material. From this we were led to believe that our profession was recognized by the Government, and that Dentists were appointed in the army and navy. Shortly after, however, when General Sherman and his hundred thousand men entered Savannah, we were by practical demonstration better informed. From daylight until dark our dental offices were besieged. The cry was, relief from present suffering, "Do something for my teeth that will keep them from aching." We remember one fellow coming to us a few days after having one or two nerves destroyed in his teeth, who wished, as he said, "to buy a good smart quantity of that white, creamy stuff," for, said he, "when the boys get on the march, and have the tooth-ache, I can get their last dollar with it."

But this was not the only applicant for dental material. Dental instruments were in great demand, and a dental depot would have done a smashing business in Savannah for a few days, at that time. Now, would there have been such a demand had there not been a necessity? The innumerable quantity of broken teeth and fractured jaws, produced by bungling instruments and unfamiliar hands, along with stories of rheumatic and neuralgic suffering, caused by exposed pulps and diseased teeth, which we were obliged to listen to during the sojourn of the army in Savannah, were disheartening; and we determined then, should an opportunity ever occur, to contribute our mite towards a remedy.

We can not but think our General Medical Director was remiss in his duty in this respect; but not as much so as the members of our own profession, whose duty it was to properly bring the matter before him. At the North almost every State had its society. The American Association and American Convention were in full operation; besides three or four Dental colleges, and as many Dental journals; yet with all this array of strength and talent we hear of little or no effort being made for the end in view. We believe, had the subject been properly brought to the notice of the General Medical Director, and with his aid before Congress, by some one of our societies, that dental appointments would have been made in the army and navy; and that the good developed by such appointments would, before now, have produced full recognition on the part of our Government, as a necessity; and that to-day American Dentistry would be occupying a much more elevated position in the eyes of the world than it does.

With us in the South it was very different. We had no dental society or journal, and owing to the irregularity of the mails, and stringency of conscription, we could not act collectively but had to do what we could individually. As the war went on, and the call for men was renewed and the conscription increased to fifty, we were thinned to almost a corporal's guard. Many towns, of several thousand inhabitants, had no Dentist. This state of affairs wrought a change little thought of. Officers and government employees, including their families, found the inconvenience and suffering too great to be endured, and their appeals became so numerous and urgent that the Surgeon General was obliged to recommend the detailing of Dentists for many towns and hospitals. The hospital practice was productive of so much good that the appointment of a Dentist to each regiment was strongly urged, as a means of increasing the active duty list of the army. The following is an extract from a memorial which was presented about that time:

"Our own experience with soldiers, in and around Richmond during the last year, in connection with the statements of some of the most intelligent physicians and officers in the service, fully convinces us that out of every one hundred men sent to the hospital, or those on the sick list—exclusive of those wounded in battle—five, at least can be traced directly, or indirectly, to some derangement of the teeth; that might have been remedied in a few minutes, or hours. Thus we have of one hundred thousand men *five thousand* unnecessarily off duty. Now, this five thousand in every hundred thousand, might be returned to active duty, or prevented from leaving it, by the appointment of a few Dentists, say one to every army division."

This, no doubt, would have had its effect, and appointments would have been made, but the end came, and the project was lost with "the lost cause."

Now, is it not humiliating to our nation and profession to be obliged to acknowledge that the English, with their better teeth and worse dentistry, have exercised more humanity in this matter than we have? Just think of one hundred thousand men, as in the case of the army of General Sherman between Atlanta and Savannah, exposed for months to all kinds of weather, and imagine the amount of suffering they must have endured, owing to the want of a few Dentists! The army of Washington can not be argued against this, for then our teeth were much better, and no more what they are now than was the practice of Dentistry. Again, it can not be argued that as the war is over the necessity is past; for every now and then we hear of war vessels leaving for some distant port, with from three to five hundred men, who are away for months and years, without ever seeing a Dentist.

We all know how and wherein the army and navy could be benefited by the appointment of Dentists; but it is no easy matter to convince the appointing power. No doubt, could we make them look on the broken teeth and fractured jaws, and force them to listen to the stories of suffering, above mentioned; and better still, experience a little of the pain there alluded to, away from the means of relief, it would not be long before this vacuum in our army and navy would be filled. But as we can not do this we must do what is next best, we must keep on telling them until they will believe us. No military school; no army post, or station; no naval ship, or foreign port where ships are stationed, should be without a dentist. Our sailors and soldiers are no more able to pay for dental operations than they are for medical attention; therefore, if we value their services, we should, as a humane and enlightened nation, value their lives. In many distant and foreign ports our sailors and representatives are no better off than at sea. Take the ports of Japan, for instance, where the native surgeons extract teeth by giving them a thorough malleting on both sides, until they are loose enough to be removed. The field for good to our nation and profession in such ports is extensive. The advanced state of dentistry would necessarily (as has been the case in Paris with Evans, and others) bring us in contact with the officials and higher classes of the country; and we would thus reach influential ears that are now closed to us. But the most direct and greatest good would be to our sailors and representatives, stationed there; for they would thus be within reach of innumerable advantages, which the advanced state of dentistry offers. To oblige the army and navy surgeons to fill this vacuum is wrong; it is inhuman, for with their medical education only they are little better prepared to perform any dental operation than the patients they attempt to relieve. No medical college has made any attempt to educate their students in this specialty for the last twenty years, they have given it over to the dental schools, where it properly belongs. Then modern literature is silent on this matter, and not enough can be found in the older works to pay the trouble of research. What physician, or who else would think of going to a medical man for any disease of his teeth? Why then the sailor or soldier? Army and navy surgeons of any intelligence know that, being obliged to perform these operations, they are placed at great disadvantage, and with thankful hearts would hail relief. But even were this not so, we should not be detered; for feeling and knowing we are right we should go ahead until the object is obtained. We may encounter (as has been the case with every new undertaking) a few wise owls, who blink and scowl at every ray of light that comes within range. There were many such in the days of Galileo and Harvey, and we fear there are yet a few left. But with due honor to our parent tree, the medical profession, we believe at the present day they are not over encumbered with this class, and judging from the past, and what we know of the present we think we may depend on their sympathy and co-operation in this our new enterprise for alleviating the sufferings of humanity.

